# Appendicitis-Like Picture Induced by Foreign Body in a 2-Year-Old Boy

**DOI:** 10.1155/2020/8877754

**Published:** 2020-12-24

**Authors:** Sadi A. Abukhalaf, Rami A. Misk, Hussam I. Alzeerelhouseini, Ismaeel M. Irziqat, Abdulrahman H. Asaferah, Osama Y. Aljabarein, Khalil N. Abuzaina

**Affiliations:** ^1^Faculty of Medicine, Al Quds University, Jerusalem, State of Palestine; ^2^Faculty of Medicine and Health Sciences, Palestine Polytechnic University, Hebron, State of Palestine; ^3^Governmental Hebron Hospital, Hebron, State of Palestine

## Abstract

*Background and Aim*. Appendicitis is unusual in toddlers and foreign body- (FB-) induced appendicitis is rare. We present a FB-induced appendicitis in a toddler with no suggestive history of FB ingestion. *Case Presentation*. A 2-year-old healthy boy presented to the emergency department with irritability for 3 days duration associated with fever of 39°C, nausea, anorexia, and vomiting. There was no history of foreign body ingestion. The abdomen was distended and diffusely tender. An abdominal ultrasound (US) was suggestive of perforated appendicitis with appendicular mass formation. An abdominal X-ray showed a pin-like foreign body in the abdomen. An emergent appendectomy was performed. Intraoperatively, a sealed small cecal perforation was noticed. A 5 cm pin-like metallic foreign body was found to obstruct the appendicular lumen. The appendix was grossly normal without inflammatory changes. *Conclusion*. FB-induced perforations or appendicitis albeit in patients with no history of FB ingestion or infants and toddlers need a high clinical suspicion to prevent the delay in diagnosis and the subsequent complications.

## 1. Introduction

Appendicitis is one of the most common causes of surgical acute abdomen in children though it is most common during the second decade of life, and it is uncommon in preschool children. Foreign body- (FB-) induced appendicitis is one of the uncommon causes of appendicitis with an estimated prevalence of 0.05%. FB may remain in the appendix without any complication or cause inflammation of the appendix with or without perforation [1–3]. Perforation of the gastrointestinal tract by FB is rare with an incidence rate of less than 1%. Patients may have a delay in the diagnosis and present late with complications as perforation and peritonitis especially in patients with absence of any history for foreign body ingestion, atypical age, and/or nonspecific presentation [4–6]. Herein, we present a 2-year-old boy with no suggestive history of foreign body ingestion presented with nonspecific symptoms and turned to have FB-induced appendicitis

## 2. Case Presentation

A 2-year-old healthy boy presented to the emergency department with irritability for 3 days duration associated with fever of 39 C, nausea, anorexia, and vomiting. Vital signs were within normal limits except for a fever of 38°C. There was no history of foreign body ingestion. On examination, the child was irritable and pointing to the right lower area of the abdomen. The abdomen was distended and diffusely tender on palpation with localized rigidity at the right iliac fossa (RIF). Laboratory tests showed a white cell count of 24,300 cells/mm^3^ with a neutrophil predominance of 18,000 cells/mm^3^ and normal urine analysis.

**Figure 1:**
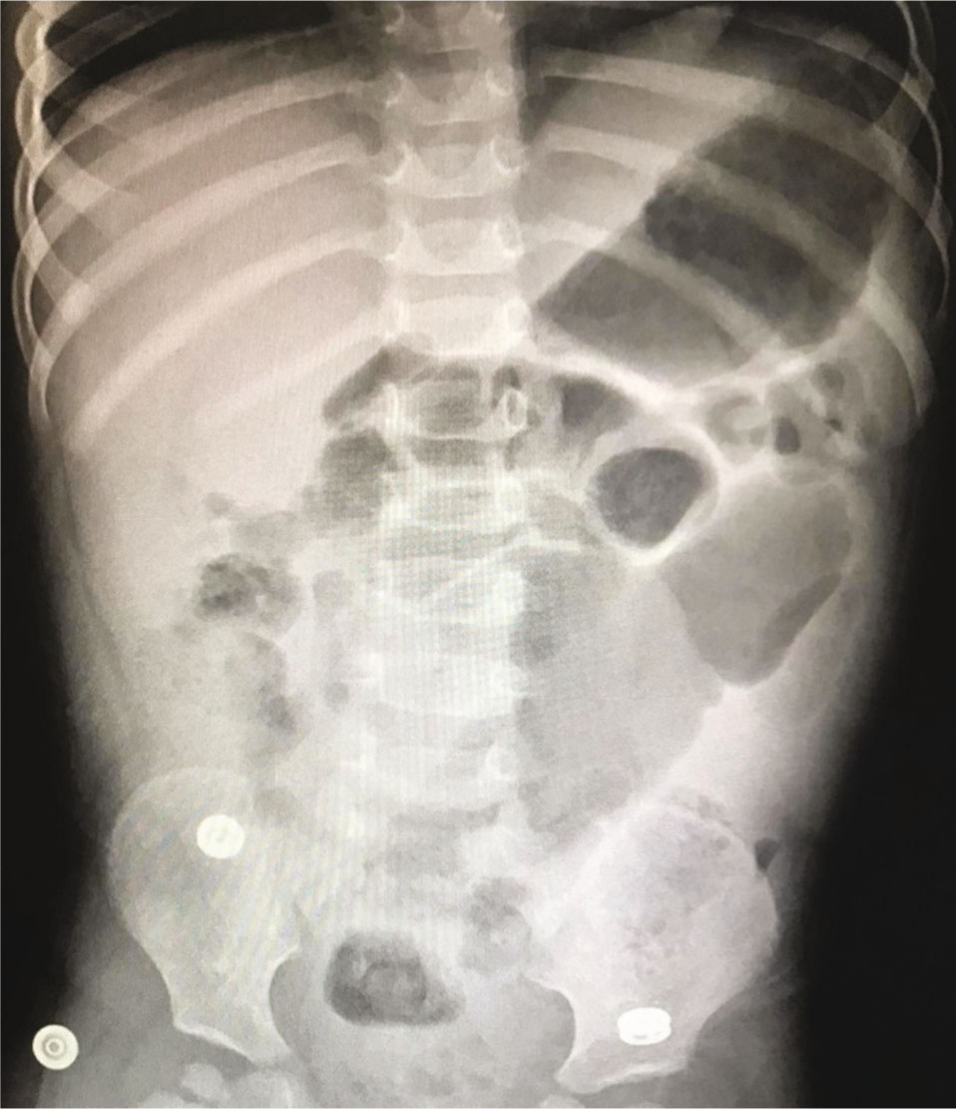
Preoperative abdominal X-ray shows foreign body ingestion though there was no suggestive history.

An abdominal ultrasound (US) showed that the appendix was noted at the right iliac fossa measuring about 8.5 mm in diameter surrounded by inflammatory process manifested by echogenic fatty planes with edematous mesentery and enlarged mesenteric lymph nodes with mild pelvic free fluid suggestive of perforated appendicitis with appendicular mass formation. An abdominal X-ray showed a pin-like foreign body in the abdomen (Figure 1).

**Figure 2:**
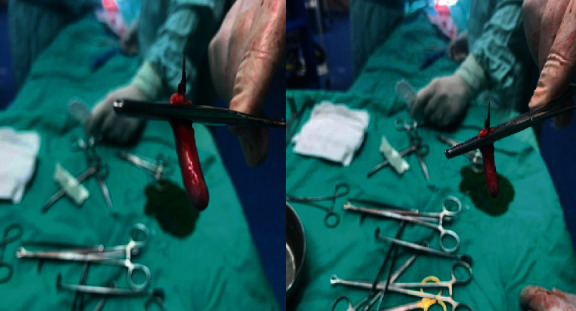
Metallic foreign body that obstructs the appendiceal lumen.

**Figure 3:**
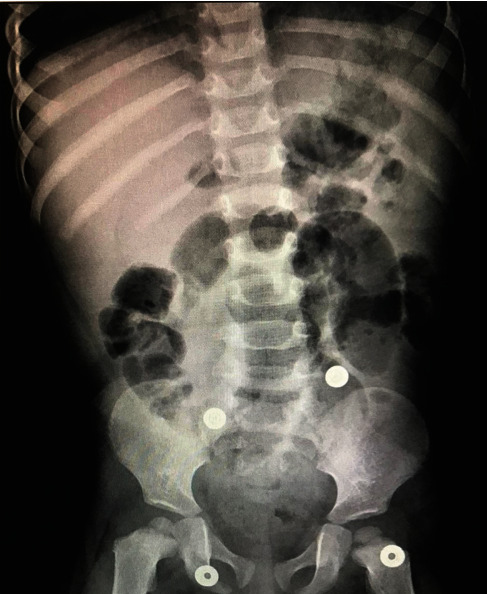
Postoperative abdominal X-ray shows resolution of the foreign body previously found preoperatively.

The patient underwent an emergent appendectomy. Intraoperatively, the distal omentum was found to surround a small area of the cecum with minimal necrosis seen at its surface which may represent a sealed small cecal perforation. Although the appendix was grossly normal, the surgeon had decided to perform an appendectomy. Surprisingly, a 5 cm pin-like metallic foreign body was found to obstruct the appendicular lumen (Figure 2). The specimen was sent for histopathology analysis, which revealed serosal inflammation not involving the mucosa (periappendicitis) with fibrous obliteration of the appendicular lumen. On postoperative day (POD) 3, the patient was discharged home though he came back due to irritability (Figure 3). An abdominal US showed echogenic fatty planes at RIF. At POD 6, another abdominal US showed mild to moderate intraabdominal fluids with echogenic fatty planes and multiple enlarged mesenteric lymph nodes at the RIF but with no evidence of collection. The patient was diagnosed with paralytic ileus. However, the patient's condition started to get better and was discharged home on POD 9.

## 3. Discussion

Acute abdominal pain is one of the most common complaints in children; this includes a wide range of surgical and nonsurgical conditions, which vary with age, associated symptoms, and pain location. Although acute abdominal pain in children is usually due to benign self-limiting causes such as gastroenteritis, several threatening causes may require urgent surgical intervention such as appendicitis [1].

One of the most common causes of surgical acute abdomen in children is appendicitis. However, appendicitis is most common during the second decade of life, and it is uncommon in preschool children. In a study conducted in 2012 for all children less than 5 years of age and presented with acute appendicitis over 12 years, only 5% of the patients were less than 3 years [2, 3].

Foreign body- (FB-) induced appendicitis is one of the uncommon causes of appendicitis but has been documented in the literature with an estimated prevalence of 0.05% [3, 4]. Even more than 90% of FBs pass uneventfully through the gastrointestinal tract without complications; perforation of the gastrointestinal tract by FBs is rare with less than 1% incidence rate. Different foreign bodies have been reported as the cause of appendicitis including fruit seeds, pins, needles, teeth, bone fragments, coins, stones, and toothbrush bristle [3–9].

In consideration of the dependent position of the cecum, FB tends to settle down especially the heavy FBs, and then the appendiceal orifice will expand to allow the FBs to lodge into its lumen [10–13]. However, this scenario is impossible to happen in the case of a retrocecal appendix [4].

The FB may remain in the appendix without any complication or cause inflammation of the appendix with or without perforation. The period between the FBs ingestion and the onset of symptoms may vary from hours to years [5, 7, 9]. However, the complication in such cases varies according to the size and the shape of the FB. Blunt objects tend to obstruct the appendiceal lumen and cause appendicitis, whereas sharp objects are more likely to cause perforation and appendicitis [4–6, 9].

The diagnosis of the foreign body-induced appendicitis in our patient was very challenging due to several causes: the absence of any history for foreign body ingestion, the atypical age for appendicitis, nonspecific presentations, the overlap of presentation with many other common childhood conditions, and poor compliance of the patient to the abdominal exam. Therefore, most such patients have a delay in the diagnosis and usually present late with complications, e.g., perforation and peritonitis [3, 14, 15].

Nance et al.'s study involved 120 patients who were 5 years of age or less and required an operation for appendicitis showed that 74% of the patients were found to have perforation at the time of the surgery [16]. Therefore, extensive history and physical examination in addition to a high index of suspicion are needed in such cases to have a better outcome and preventing the delay in diagnosis and complications.

Diagnostic laparoscopy for the management of a foreign body in the gastrointestinal tract as well as in the appendix is the best option and has been well described in the literature [4, 17, 18]. Laparoscopic appendectomy (LA) is well accepted as the gold standard of care in cases of acute appendicitis [19, 20]. Many studies demonstrate LA to be achievable, safe, and effective [21–24].

These studies show LA to be superior to open appendectomy (OA) due to shortened hospital stay, lower complication rates, and earlier return to normal activity [25, 26]. The higher cost for LA is one of its major limitations [27–29]. Over the last decade, a new technique, single incision laparoscopic surgery (SILS), has been developed to make more cosmetically acceptable incisions and reduce postoperative pain and the period of return to normal activity [30].

Several studies have tested and compared SILA with LA showing similar postoperative results [31, 32]. However, the increased costs for SILA compared with LA are still a major limitation to this technique [33, 34].

The surgical glove port-single incision laparoscopic appendectomy (SGP-SILA) is a new safe, achievable, and alternative to the classic SILA. Di Saverio et al.'s study has shown that SGP-SILA is more cost-effective compared to the standard SILA or even of the traditional multiport LA [35].

Conservative management may be an option especially in the case of an acute perforated appendix that formed an inflamed mass instead of free perforation though there is no strong evidence to support conservative for the foreign body in the appendix [6].

## 4. Conclusion

Foreign body-induced appendicitis is rare with an estimated prevalence of 0.05%. Children may have FB-induced appendicitis albeit there was no suggestive ingestion history. Appendicitis may occur in children of 2 years old though causes should be searched as FB. Perforated appendicitis with inflamed mass may be managed conservatively; however, FB-induced perforated appendicitis may not be managed similarly.

## References

[1] Kim J. S. (2013). Acute abdominal pain in children. *Pediatric Gastroenterology, Hepatology & Nutrition*.

[2] Addiss D. G., Shaffer N., Fowler B. S., Tauxe R. V. (1990). The epidemiology of appendicitis and appendectomy in the United States. *American Journal of Epidemiology*.

[3] Bansal S., Banever G. T., Karrer F. M., Partrick D. A. (2012). Appendicitis in children less than 5 years old: influence of age on presentation and outcome. *The American Journal of Surgery*.

[4] Klingler P., Seelig M., DeVault K. (1999). Ingested foreign bodies within the appendix: a 100-year review of the literature. *Digestive Diseases*.

[5] Song Y. S., Covarrubias D. A., Nardi P. M. (2009). Foreign body appendicitis. *American Journal of Roentgenology*.

[6] Sar S., Mahawar K. K., Marsh R., Small P. K. (2009). Recurrent appendicitis following successful conservative management of an appendicular mass in association with a foreign body: a case report. *Cases Journal*.

[7] Fischer C. D., Mukherjee A. (2004). Appendicitis due to tongue stud ingestion: a case study and review of management plans. *South Dakota Journal of Medicine*.

[8] Hazer B., Dandin O., Karakaş D. Ö. (2013). A rare cause of acute appendicitis: an ingested foreign body. *Ulusal Travma ve Acil Cerrahi Dergisi*.

[9] Benizri E. I., Cohen C., Bereder J. M., Rahili A., Benchimol D. (2012). Swallowing a safety pin: report of a case. *World Journal of Gastrointestinal Surgery*.

[10] Bababekov Y. J., Stanelle E. J., Abujudeh H. H., Kaafarani H. M. (2015). Fishbone-induced perforated appendicitis. *Case Reports*.

[11] Conti F., Gentilli S., Mauri A. (1993). Foreign body decubitus: unusual cause of acute appendicitis. *Minerva Chirurgica*.

[12] Meyer J. O. S. E. P. H., Abuabara S. A. B. A. S., Barrett J. O. H. N., Lowe R. O. B. E. R. T. (1982). A bullet in the appendix. *The Journal of Trauma*.

[13] Green S. M., Schmidt S. P., Rothrock S. G. (1994). Delayed appendicitis from an ingested foreign body. *The American Journal of Emergency Medicine*.

[14] Williams N., Kapila L. (1991). Acute appendicitis in the preschool child. *Archives of Disease in Childhood*.

[15] Barker A. P., Davey R. B. (1988). Appendicitis in the first three years of life. *Australian and New Zealand Journal of Surgery*.

[16] NANCE M. L., ADAMSON W. T., HEDRICK H. L. (2000). Appendicitis in the young child: a continuing diagnostic challenge. *Pediatric Emergency Care*.

[17] Hartin C. W., Lau S. T., Caty M. G. (2008). Metallic foreign body in the appendix of 3-year-old boy. *Journal of Pediatric Surgery*.

[18] Mehran A., Podkameni D., Rosenthal R., Szomstein S. (2005). Gastric perforation secondary to ingestion of a sharp foreign body. *JSLS: Journal of the Society of Laparoendoscopic Surgeons*.

[19] Heinzelmann M., Simmen H. P., Cummins A. S., Largiadèr F. (1995). Is laparoscopic appendectomy the new ‘gold standard’?. *Archives of Surgery*.

[20] Kehagias I., Karamanakos S. N., Panagiotopoulos S., Panagopoulos K., Kalfarentzos F. (2008). Laparoscopic versus open appendectomy: which way to go?. *World Journal of Gastroenterology*.

[21] Huang M. T., Wei P. L., Wu C. C., Lai I. R., Chen R. J., Lee W. J. (2001). Needlescopic, laparoscopic, and open appendectomy: a comparative study. *Surgical Laparoscopy, Endoscopy & Percutaneous Techniques*.

[22] Ignacio R. C., Burke R., Spencer D., Bissell C., Dorsainvil C., Lucha P. A. (2004). Laparoscopic versus open appendectomy: what is the real difference? Results of a prospective randomized double-blinded trial. *Surgical Endoscopy*.

[23] Di Saverio S., Mandrioli M., Sibilio A. (2014). A cost-effective technique for laparoscopic appendectomy: outcomes and costs of a case-control prospective single-operator study of 112 unselected consecutive cases of complicated acute appendicitis. *Journal of the American College of Surgeons*.

[24] Mandrioli M., Inaba K., Piccinini A. (2016). Advances in laparoscopy for acute care surgery and trauma. *World Journal of Gastroenterology*.

[25] Ohtani H., Tamamori Y., Arimoto Y., Nishiguchi Y., Maeda K., Hirakawa K. (2012). Meta-analysis of the results of randomized controlled trials that compared laparoscopic and open surgery for acute appendicitis. *Journal of Gastrointestinal Surgery*.

[26] Wei B., Qi C. L., Chen T. F. (2011). Laparoscopic versus open appendectomy for acute appendicitis: a metaanalysis. *Surgical Endoscopy*.

[27] Harrell A. G., Lincourt A. E., Novitsky Y. W. (2006). Advantages of laparoscopic appendectomy in the elderly. *The American Surgeon*.

[28] McCahill L. E., Pellegrini C. A., Wiggins T., Helton W. S. (1996). A clinical outcome and cost analysis of laparoscopic versus open appendectomy. *American Journal of Surgery*.

[29] Sauerland S., Jaschinski T., Neugebauer E. A. (2010). Laparoscopic versus open surgery for suspected appendicitis. *Cochrane Database of Systematic Reviews*.

[30] Pelosi M. A., Pelosi M. A. (1992). Laparoscopic appendectomy using a single umbilical puncture (minilaparoscopy). *The Journal of Reproductive Medicine*.

[31] Hua J., Gong J., Xu B., Yang T., Song Z. (2014). Single-incision versus conventional laparoscopic appendectomy: a meta-analysis of randomized controlled trials. *Journal of Gastrointestinal Surgery*.

[32] Markar S. R., Karthikesalingam A., Di Franco F., Harris A. M. (2013). Systematic review and meta-analysis of single-incision versus conventional multiport appendicectomy. *British Journal of Surgery*.

[33] Chow A., Purkayastha S., Nehme J., Darzi L. A., Paraskeva P. (2010). Single incision laparoscopic surgery for appendicectomy: a retrospective comparative analysis. *Surgical Endoscopy*.

[34] St Peter S. D., Adibe O. O., Juang D. (2011). Single incision versus standard 3-port laparoscopic appendectomy: a prospective randomized trial. *Annals of Surgery*.

[35] Di Saverio S., Mandrioli M., Birindelli A. (2016). Single-incision laparoscopic appendectomy with a low-cost technique and surgical-glove port: “how to do it” with comparison of the outcomes and costs in a consecutive single-operator series of 45 cases. *Journal of the American College of Surgeons*.

